# Modulation of Gut Microbiota: A Novel Paradigm of Enhancing the Efficacy of Programmed Death-1 and Programmed Death Ligand-1 Blockade Therapy

**DOI:** 10.3389/fimmu.2018.00374

**Published:** 2018-03-05

**Authors:** Yiming Wang, Rena Ma, Fang Liu, Seul A. Lee, Li Zhang

**Affiliations:** ^1^School of Biotechnology and Biomolecular Sciences, University of New South Wales, Sydney, NSW, Australia

**Keywords:** gut microbiota, programmed death 1, programmed death ligand 1, cancer immunotherapy, efficacy

## Abstract

Blockade of programmed death 1 (PD-1) protein and its ligand programmed death ligand 1 (PD-L1) has been used as cancer immunotherapy in recent years, with the blockade of PD-1 being more widely used than blockade of PD-L1. PD-1 and PD-L1 blockade therapy showed benefits in patients with various types of cancer; however, such beneficial effects were seen only in a subgroup of patients. Improving the efficacy of PD-1 and PD-L1 blockade therapy is clearly needed. In this review, we summarize the recent studies on the effects of gut microbiota on PD-1 and PD-L1 blockade and discuss the new perspectives on improving efficacy of PD-1 and PD-L1 blockade therapy in cancer treatment through modulating gut microbiota. We also discuss the possibility that chronic infections or inflammation may impact on PD-1 and PD-L1 blockade therapy.

## Introduction

The immune system uses various effector cells and molecules to control and eradicate infectious agents and cancer cells. Cytotoxic T cells (CTL) are the major effector cells in anti-tumor immune responses (1, 2). However, the functions of these effector cells are inhibited in the tumor microenvironment, which contributes to cancer cell immune evasion (3). In recent years, the blockade of immune checkpoint proteins and molecules that deliver inhibitory signals to activated T cells, have shown great promise in cancer treatment. However, the beneficial effects of these treatment strategies were seen only in a subgroup of patients (4). In this review, we summarize the emerging evidence of improving immune checkpoint protein blockade therapy efficacy by modulating gut microbiota and discuss the possibility that chronic infections or inflammation may impact on programmed death 1 (PD-1) and programmed death ligand 1 (PD-L1) blockade therapy.

## PD-1 and Its Ligands

Programmed death 1, also known as cluster of differentiation 279 (CD279), is a cell surface receptor that was discovered in 1992 (5). PD-L1 and PD-L2, the two molecules that interact with PD-1, were identified in the following years (6, 7). PD-L1 is also known as CD274 or B7 homolog 1 (B7-H1) and PD-L2 known as CD273 or B7-DC.

Programmed death 1 is expressed on T, B cells, and myeloid cells (8). PD-L1 is expressed by a variety of cells in the immune system and non-immune cells. However, the expression level of PD-L1 in normal human tissues is low; despite the presence of PD-L1 mRNA, PD-L1 protein is rarely detected on the cell surfaces in most of normal human tissues except for a subset of human tissue macrophages (6, 9). PD-L2 is predominately expressed by antigen-presenting cells, such as dendritic cells (DCs) and macrophages (10–13). The expression of both PD-L1 and PD-L2 is regulated by cytokines, for example interferon (IFN)-γ greatly increases the expression of PD-L1 and several cytokines are able to induce the expression of PD-L2 in other immune cells and non-immune cells in addition to the DCs and macrophages (9–11, 14–16).

Programmed death 1 and its ligands are members of the immune checkpoint proteins delivering inhibitory signals to activated T cells. The interaction of PD-1 with PD-L1 or PD-L2 leads to suppression of T cells, a regulatory mechanism to prevent overstimulation of immune responses and autoimmunity (6, 7, 9, 16–21). However, such a mechanism is hijacked in the tumor microenvironment, providing opportunities for tumor cells to evade the attack from the immune system.

## PD-1 and PD-L1 Blockade in Cancer Immunotherapy

In anti-tumor immune responses, the tumor antigens generated by gene mutations, are recognized by the immune system and specific CD8+ CTLs targeting tumor antigens are generated (22). These specific effector CTLs recognize the target tumor cells and induce tumor cell apoptosis.

However, tumor cells employ various strategies to escape the attack from the immune system, one of which is to resist the killing effects from the anti-tumor CTLs by increasing PD-L1 expression in tumor tissues (9, 23, 24). Most normal human tissues do not express detectable PD-L1 on their cell surface, in contrast PD-L1 is abundantly expressed by tumor cells, the immune and non-immune cells in various tumor tissues (6, 9, 25–30). IFN-γ released by the anti-tumor CTLs infiltrating into tumor tissues plays a major role in inducing the expression of PD-L1 (9–11, 14–16). Other cytokines, such as tumor necrosis factor (TNF) -α, interleukin (IL)-4, and IL-10 can also increase PD-L1 expression (31, 32).

**Figure 1:**
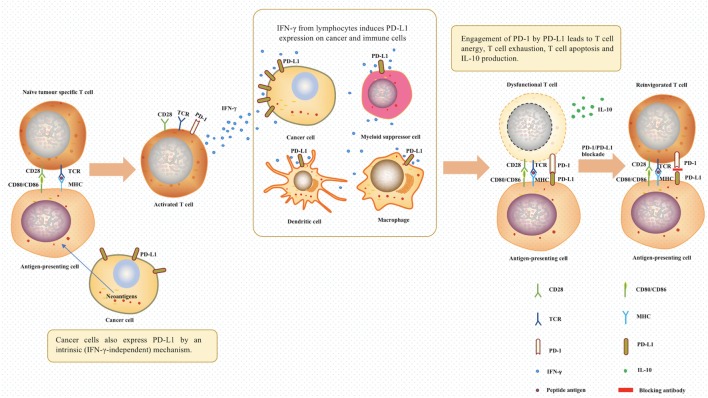
The role of programmed death 1 (PD-1) and programmed death ligand 1 (PD-L1) in tumor evasion and cancer immunotherapy. In the tumor microenvironment, T cells were activated after antigen-presenting cells recognized tumor neoantigens. The IFN-γ produced by activated T cells induced the expression of PD-1 ligands on cancer cells and immune cells. Afterward, the engagement of PD-1 by PD-L1 between T cells and antigen-presenting cells will lead to T cell dysfunction. PD-1/PD-L1 blockade using relevant antibodies can inhibit this process, therefore, offering a chance for T cells to continue being effectors. Abbreviations: TCR, T-cell receptor; MHC, major histocompatibility complex; IFN-γ, interferon gamma; IL-10, interleukin 10.

The interaction of PD-L1 with PD-1 in the tumor microenvir-onment enables the tumor cells to resist the endogenous anti-tumor activities from the immune system. PD-L1 expressed in tumor tissues interacting with PD-1 expressed on the activated T cells leads to the dysfunction of the effector T cells, *via* multiple mechanisms, such as promoting T cell apoptosis, anergy, and exhaustion (6, 7, 9, 16–21). More recently, it was found that interaction of PD-L1 with PD-1 expressed on tumor-associated macrophages inhibits the phagocytic potency of macrophages against tumor cells (33). The importance of PD-L1 and PD-1 interaction in tumor cell evasion has led scientists to explore the use of these molecules as therapeutic targets in cancer immunotherapy (Figure 1).

Dong et al. showed that PD-L1 positive human tumor cells induced apoptosis of co-cultured activated effector T cells and this effect was blocked by an anti-human PD-L1 monoclonal antibody (mAb). They also showed that the growth of PD-L1 positive murine tumors in syngeneic mice was suppressed by an anti-murine PD-L1 mAb (9). Other researchers later reported similar findings in examination of different types of cancer cells using mice models (24, 34–36). These important laboratory observations led to numerous clinical trials of using monoclonal antibodies targeting PD-1 or PD-L1 in cancer immunotherapy for a variety of cancers. In addition to affecting the immunological pathways, PD-L1 and PD-1 blockade may also work in part by disrupting autologous PD-1 and PD-L1 signaling within tumors (37, 38).

To date, the U.S. Food and Drug Administration (FDA) has approved the use of five monoclonal antibodies targeting PD-L1 or PD-1 in cancer treatment. The details of the clinical trials of these five monoclonal antibodies are summarized in Table 1. Despite the clear benefits of PD-L1 and PD-1 blockade in treating some cancer patients, not all cases responded to treatment (Table 1). Given this, strategies to improve the efficacy of cancer immunotherapy are needed. Emerging evidence suggests that modulation of the gut microbiota is a promising approach.

**Table 1:** Five monoclonal antibodies targeting programmed death ligand-1 (PD-L1) or programmed death 1 (PD-1) were approved by the U.S. Food and Drug Association to treat cancer.

Commercial name (active ingredient)	Target	Treatment of disease	Targeting patients	Clinical cases	Clinical phase	Overall response rate (95% CI)	Objective response rate (95% CI)	Clinical study (clinical trial ID)	Reference
Bavencio (Avelumab)	PD-L1	Metastatic MCC	Metastatic MCC patients whose disease had progressed on or after chemotherapy administered	88	Phase 2	33% (23.3%, 43.8%)	Not applicable	JAVELIN Merkel 200 Trial (NCT02155647)	(39, 40)
Tecentriq (Atezolizumab)	PD-L1	Advanced or metastatic urothelial carcinoma	Cisplatin-ineligible patients with locally advanced or metastatic urothelial carcinoma	119	Phase 2	23.5% (16.2%, 32.2%)	Not applicable	IMvigor210 (NCT02951767)	(41)
			Previously treated patients with locally advanced or metastatic urothelial carcinoma	310	Phase 2	14.8% (11.1%, 19.3%)	Not applicable	IMvigor210 (NCT02951767)	(41)
		Metastatic NSCLC	Previously treated patients with metastatic non-small cell lung cancer	22	Phase 2	Not applicable	15% (10%, 22%)	POPLAR (NCT01903993)	(42)
Imfinzi (Durvalumab)	PD-L1	Locally advanced or metastatic urothelial carcinoma	Patients with locally advanced or metastatic urothelial carcinoma in total	182	Phase 1 and 2	Not applicable	17.0% (11.9%, 23.3%)	Study 1108 (NCT01693562)	(43–45)
			Patients with locally advanced or metastatic urothelial carcinoma who showed high PD-L1 expression on tumor cells	95	Phase 1 and 2	Not applicable	26.3% (17.8%, 36.4%)	Study 1108 (NCT01693562)	(43–45)
			Patients with locally advanced or metastatic urothelial carcinoma who showed low or non-PD-L1 expression on tumor cells	73	Phase 1 and 2	Not applicable	4.1% (0.9%, 11.5%)	Study 1108 (NCT01693562)	(43–45)
Keytruda (Pembrolizumab)	PD-1	Melanoma	Patients with Ipilimumab-Naïve melanoma (receive KEYTRUDA at a dose of 10 mg/Kg every 3 weeks)	277	Phase 3	33% (27%, 39%)	Not applicable	KEYNOTE-006 (NCT01866319)	(46, 47)
			Patients with Ipilimumab-Naïve melanoma (receive KEYTRUDA at a dose of 10 mg/Kg every 2 weeks)	279	Phase 3	34% (28%, 40%)	Not applicable	KEYNOTE-006 (NCT01866319)	(46, 47)
			Patients with Ipilimumab-refractory melanoma (receive KEYTRUDA at a dose of 2 mg/Kg every 3 weeks)	180	Phase 2	Not applicable	21% (15%, 28%)	KEYNOTE-002 (NCT01704287)	(48)
			Patients with Ipilimumab-refractory melanoma (receive KEYTRUDA at a dose of 10 mg/Kg every 3 weeks)	181	Phase 2	Not applicable	25% (19%, 32%)	KEYNOTE-002 (NCT01704287)	(48)
		NSCLC	Metastatic NSCLC patients with first-line treatment with a single agent	154	Phase 3	Not applicable	45% (37%, 53%)	KEYNOTE-024 (NCT02142738)	(49)
			Metastatic NSCLC patients with first-line treatment in combination with pemetrexed and carboplatin	60	Phase 1 and 2	55% (42%, 68%)	Not applicable	KEYNOTE-021 (NCT02039674)	(50)
			Previously treated NSCLC patients (all randomized patients who receive KEYTRUDA at a dose of 2 mg/Kg every 3 weeks)	344	Phase 2 and 3	Not applicable	18% (14%, 23%)	KEYNOTE-010 (NCT01905657)	(51)
			Previously treated NSCLC patients (all randomized patients who receive KEYTRUDA at a dose of 10 mg/Kg every 3 weeks)	346	Phase 2 and 3	Not applicable	19% (15%, 23%)	KEYNOTE-010 (NCT01905657)	(51)
		HNSCC	HNSCC patients whose disease had progressed on or after chemotherapy administered	174	Phase 1	16% (11%, 22%)	Not applicable	KEYNOTE-012 (NCT01848834)	(52)
		Urothelial Carcinoma	Cisplatin-ineligible patients with urothelial carcinoma	370	Phase 2	Not applicable	29% (24%, 34%)	KEYNOTE-052 (NCT02335424)	(53)
			Previously treated urothelial carcinoma patients	270	Phase 3	Not applicable	21% (16%, 27%)	KEYNOTE-045 (NCT02256436)	(54)
		cHL	Patients with cHL	210	Phase 2	69% (62%, 75%)	Not applicable	KEYNOTE-087 (NCT02453594)	(55, 56)
		MSI-H	Patients with MSI-H or mismatch repair deficient (dMMR)	149	Phase 1 Phase 2 Phase 1 Phase 2 Phase 2	Not applicable	39.6% (31.7%, 47.9%)	KEYNOTE-012 (NCT01848834) KEYNOTE-016 (NCT01876511) KEYNOTE-028 (NCT02054806) KEYNOTE-158 (NCT02628067) KEYNOTE-164 (NCT02460198)	(52, 57–59)
Opdivo (Nivolumab)	PD-1	Unresectable or metastatic melanoma	Previously treated patients with unresectable or metastatic melanoma in the treatment of OPDIVO	316	Phase 3	Not applicable	40% (34%, 46%)	CheckMate-067 (NCT01844505)	(60, 61)
			Previously treated patients with unresectable or metastatic melanoma in the treatment of OPDIVO plus Ipilimumab (anti-CTLA4 antibody)	314	Phase 3	Not applicable	50% (44%, 55%)	CheckMate-067 (NCT01844505)	(60, 61)
		Metastatic NSCLC	NSCLC patients who had experienced disease progressed during or after one prior platinum doublet-based chemotherapy regimen	272	Phase 3	Not applicable	20% (14%, 28%)	CheckMate-017 (NCT01642004)	(62)
			Patients with metastatic non-squamous NSCLC who had experienced disease progressed during or after one prior platinum doublet-based chemotherapy regimen	292	Phase 3	Not applicable	19% (15%, 24%)	CheckMate-057 (NCT01673867)	(63)
		Renal cell carcinoma	Patients with advanced RCC who had experienced disease progressed during or after one or two prior anti-angiogenic therapy regimes	410	Phase 3	Not applicable	21.5% (17.6%, 25.8%)	CheckMate-025 (NCT01668784)	(64, 65)
		cHL	Adult patients with cHL	258	Phase 2 Phase 1	Not applicable	69% (63%, 75%)	CheckMate-205 (NCT02181738) CA209-039 (NCT01592370)	(66, 67)
		Recurrent or metastatic SCCHN	Patients with metastatic or recurrent SCCHN	240	Phase 3	Not applicable	13.3% (9.3%, 18.3%)	CheckMate-141 (NCT02105636)	(68, 69)

*Five monoclonal PD-L1 or PD-1 antibodies granted after May 2017 by FDA for cancer treatments were not included in the table*.

*MCC, metastatic Merkel cell carcinoma; NSCLC, non-small cell lung cancer; HNSCC, head and neck squamous cell cancer; cHL, classical Hodgkin lymphoma; MSI-H, microsatellite instability-high cancer; dMMR, mismatch repair deficient; SCCHN, recurrent or metastatic squamous cell carcinoma of the head and neck*.

## Modulation of Gut Microbiota Enhances the Anti-Tumor Efficacy of PD-1 and PD-L1 Blockade Therapy

A very interesting study by Sivan et al. provided strong evidence that the efficacy of PD-L1 blockage therapy can be improved by the modulation of gut microbiota (70). In this study, Sivan et al. examined the subcutaneous growth of B16.SIY melanoma in genetically similar C57BL/6 mice raised in the Jackson Laboratory (JAX) and Taconic Farms (TAC), and found that the tumor growth was more aggressive in TAC mice as compared to that in JAX mice and that TAC mice had a significantly lower intratumoral CD8+ T cell accumulation. They then conducted various experiments, which demonstrated that gut microbiota contributed to this difference.

They first showed that prophylactic transfer of fecal material from JAX mice to TAC mice was sufficient to delay tumor growth. To examine whether microbial community alone was effective as a therapy, they administered fecal material from JAX mice alone or in combination with anti-PD-L1 mAbs to TAC mice. These experiments showed that fecal material alone was sufficient to significantly inhibit tumor growth and that the combination treatment further improved tumor control. To identify the responsible bacterial species, they used 16S ribosomal RNA (16S rRNA) sequencing and identified *Bifidobacterium* species, particularly *Bifidobacterium breve, Bifidobacterium longum*, and *Bifidobacterium adolescentis* as the candidate species. The role of these *Bifidobacterium* species in enhancing protective immunity against tumors were further investigated by administering TAC mice bearing established tumors with a cocktail of *Bifidobacterium* species containing *B. breve* and *B. longum* by oral gavage. This experiment resulted in *Bifidobacterium*-treated mice having significantly improved tumor control as compared to mice that did not receive *Bifidobacterium*. Sivan et al. also showed that the possible mechanisms by which *Bifidobacterium* species inhibited tumor growth were through activating DCs, which in turn, improves the effector function of tumor-specific CD8+ T cells. Given that the enhanced anti-melanoma effect from *Bifidobacterium* species had occurred at the innate immunity level, the authors anticipated that *Bifidobacterium* species also provide anti-tumor beneficial effects to other types of tumors. However, the mechanisms by which *Bifidobacterium* species activated DCs improved the effects of anti-tumor CD8+ cells still need to be clarified.

**Figure 2:**
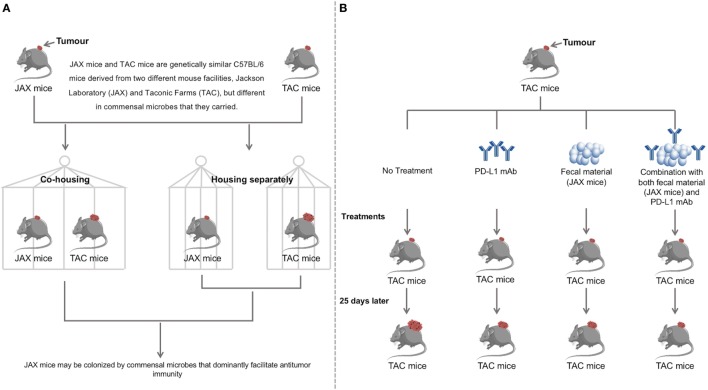
Discovery and validation of the therapeutic significance of commensal microbiota by facilitating anti-programmed death ligand-1 (PD-L1) efficacy. **(A)** Two genetically similar mice, JAX and TAC, differing in commensal microbes carried were cohoused, while another pair was housed separately. Cohousing resulted in the TAC mice obtaining the JAX microbial phenotype, with reduced tumor growth as compared to the TAC mice housed separately. JAX mice had no differences in tumor size when cohoused with TAC mice compared to separate housing. This suggests that JAX mice are colonized by commensal microbes that facilitate anti-tumor immunity. **(B)** TAC mice were treated with PD-L1 mAb, JAX mice fecal material, both the PD-L1 mAb and JAX mice fecal material or not treated. Administration of JAX fecal material alone resulted in slower tumor growth to the same degree as treatment with PD-L1 mAb. Combination treatment with both the PD-L1 mAb and JAX fecal material showed the slowest tumor growth, indicating that commensal microbes play a therapeutic role in anti-tumor immunity. Data were adapted from Sivan et al (38). Further findings in this study have demonstrated that *Bifidobacterium* is the responsible bacterial species that contributes to improving the efficacy of PD-L1 blockade therapy.

The findings by Sivan et al. using mice models suggest that it is possible to enhance the anti-tumor efficacy of PD-L1 blockade therapy in treating cancer patients by modulating their gut microbiota and their findings are summarized in Figure 2. Interestingly, a very recent study by Matson et al. examining the stool samples collected from patients with metastatic melanoma before anti-PD-1 immunotherapy found that *B. longum, Collinsella aerofaciens*, and *Enterococcus faecium* were more abundant in the anti-PD-1 immunotherapy responders, supporting the anti-tumor effects of *Bifidobacterium* species (71).

Several additional studies also compared the gut microbiota in patients with metastatic melanoma receiving anti-PD-1 therapy. A recent study by Frankel et al. using metagenomic shotgun sequencing method showed that melanoma patients who responded to immune checkpoint inhibitors were enriched with *Bacteroides caccae* (72). Furthermore, they showed that the bacteria that are enriched within responders are most likely to be antibody dependent. Patients who responded to nivolumab (PD-1 antibody) were enriched with *Fecalibacterium prausnitzii, Bacteroides thetaiotamicron*, and *Holdemania filiformis*, whereas patients who responded to pembrolizumab (another PD-1 antibody), their gut microbiota enriched with *Dorea formicogenerans*. However, the mechanisms responsible for these changes are not clear. Studies comparing the gut microbiota changes prior to and following anti-PD-1 therapy of individual patients are required, which will provide information regarding whether anti-PD-1 antibodies directly affect gut bacterial species.

A study by Wargo et al. examined the human gut microbiota and metabolites of metastatic melanoma patients who received anti-PD-1 therapy using 16S rRNA and whole genome shotgun sequencing (73). They found that bacterial diversity and composition in patients that responded to the therapy were significantly different from that in patients who did not respond to the therapy. The responding patients had a higher diversity of bacteria and a higher abundance of *Clostridiales*, and the non-responders had a higher abundance of *Bacteroidales*. In a very recent study with multiple first authors and J. A. Wargo being the responding author, they further compared the gut microbiota of patients with metastatic melanoma receiving anti-PD-1 therapy (74). They found that patients who responded to anti-PD-1 therapy were associated with a significantly higher bacterial diversity and abundance of bacteria from the *Ruminococcaceae* family, which belongs to the *Clostridiales* order, as compared to patients who did not respond to the therapy. Furthermore, they performed fecal microbiota transplantation experiments in germ-free mice, in which they showed that germ-free mice transplanted with stool samples from patients who responded to anti-PD-1 and anti-PD-L1 therapy had a significantly reduced tumor growth and improved responses to anti-PD-1 and anti-PD-L1 therapy, coupled with a higher density of CD8+ T cells. However, it is not clear which bacterial species in the *Ruminococcaceae* family has played the role in enhancing the PD-1 blockade therapy.

Another recent study by Routy et al. investigated the effects of gut microbiota in PD-1 blockade therapy (75). In their study, data from 140 patients with advanced non-small-cell-lung cancer, 67 patients with renal cell carcinoma, and 42 patients with urothelial carcinoma were collected, and they found that 69 patients who took antibiotics before or soon after starting the PD-1 blockade therapy had shorter progression-free survival and overall survival. They then explored the composition of the gut microbiota using shotgun sequencing, which showed that *Akkermansia muciniphila* was enriched in patients who responded to anti-PD-1 therapy. This suggests that *A. muciniphila* may enhance patient response to PD-1 blockade therapy. They verified this observation by transplanting the patients stool samples in specific pathogen-free mice or germ-free mice and observed tumor growth in these mice. They also found that *A. muciniphila* alone was able to restore the anti-tumor effects of PD-1 blockade that was inhibited by antibiotics. However, the mechanism by which *A. muciniphila* enhancing PD-1 blockade therapy is not known.

Bacterial species that are positively associated with PD-1 and PD-L1 blockade therapy are summarized in Table 2. Some bacterial species have also been demonstrated to affect CTLA-4 blockade immunotherapy, which were not reviewed here (76, 77).

**Table 2:** Bacterial species that are positively associated with programmed death 1 (PD-1) and programmed death ligand 1 (PD-L1) blockade therapy.

Bacteria	Model	Methods	Main findings	Reference
*Bifidobacterium breve, Bifidobacterium longum, Bifidobacterium adolescentis*	Mouse	Fecal transplantationMicrobial DNA analysisBacterial administrationCell sortingGene expression profiling	Some *Bifidobacterium* species enhanced the efficacy of anti-PD-L1 therapy *in vivo*	(70)
*Fecalibacterium prausnitzii, Bacteroides thetaiotamicron, Holdemania filiformis, Dorea formicogenerans*	Human	Metagenomic shotgun sequencingGut metabolomic profiling	Melanoma patients who responded to *nivolumab* (PD-1 antibody) were enriched with *F. prausnitzii, B. thetaiotamicron*, and *H. filiformis* Melanoma patients who responded to pembrolizumab (another PD-1 antibody), their gut microbiota enriched with *D. formicogenerans*	(72)
*Clostridiales*	Human	16S rRNA gene sequencingWhole genome shotgun sequencingImmunohistochemistryFlow cytometryCytokines assayGene expression profiling	Melanoma patients who responded to anti-PD-1 therapy had a higher diversity of bacteria and a higher abundance of *Clostridiales*	(73)
*Ruminococcaceae*^a^	MouseHuman	16S rRNA gene sequencingWhole genome shotgun sequencingImmunohistochemistryFlow cytometryCytokines assayGene expression profilingFecal microbiota transplantation	Melanoma patients who responded to anti-PD-1 therapy had a higher diversity of bacteria and a higher abundance of *Ruminococcaceae* Germ-free mice transplanted with stool samples from patients responded to anti-PD-1 and anti-PD-L1 therapy had a significantly reduced tumor growth and improved responses to anti-PD-1 and anti-PD-L1 therapy coupled with higher density of CD8+ T cells in tumor	(74)
*Akkermansia muciniphila*	MouseHuman	Metagenomic shotgun sequencingFecal microbiota transplantationImmunohistochemistryFlow cytometryCytokines assay	27% cancer patients^b^ who took antibiotics before or soon after starting the PD-1 blockade therapy had shorter progression-free survival and overall survival *A. muciniphila* was found enriched in those patients who respond to anti-PD-1 therapy *A. muciniphila* alone was able to restore the anti-tumor effects of PD-1 blockade that was inhibited by antibiotics.	(75)
*B. longum, Collinsella aerofaciens, Enterococcus faecium*	MouseHuman	16S rRNA gene sequencingMetagenomic shotgun sequencingSpecies-specific quantitative PCRImmunohistochemistryFecal transplantation	Melanoma patients who responded to anti-PD-1 therapy had a higher abundance of *B. longum, C. aerofaciens*, and *E. faecium* Germ-free mice transplanted with fecal material from responding patients could lead to improved tumor control, augmented T cell responses, and greater efficacy of anti-PD-L1 therapy	(71)

*^a^Bacteria of Ruminococcaceae family belongs to the Clostridiales order*.

*^b^Patients here include patients with advanced non-small-cell-lung cancer, renal cell carcinoma, and urothelial carcinoma*.

## Potential Mechanisms of Gut Microbes on Improving the Efficacy of PD-1 and PD-L1 Blockade Therapy

Despite the exciting findings in this research field, the underlying molecular mechanisms by which the identified gut bacterial species in the above studies enhance PD-1 and PD-L1 blockade therapy remain largely unknown.

Recently, unmethylated CpG oligodeoxynucleotides, which are abundant in bacterial DNA, were found to enhance CD8+ T cell anti-tumor immunity by downregulating PD-1 expression *via* the IL-12 pathway, suggesting that gut bacterial species that are positively associated with PD-1 and PD-L1 blockade therapy may release components that directly downregulate PD-1 or PD-L1 expression (78, 79).

It is also possible that the gut bacterial species indirectly affect PD-1 and PD-L1 expression through locally or systematically regulating immune responses, thereby affecting the efficacy of PD-1 and PD-L1 blockade therapy. Gut microbiota has been shown to impact on both innate and adaptive immune cells. Germ-free animals had a reduced number of intestinal DCs and administration of *Escherichia coli* in these animals was able to recruit sufficient DCs to the intestines (80, 81). In Germ-free pigs, systemic circulating macrophages were also reduced and their functions were compromised (82). Germ-free mice had markedly decreased presence of lamina propria CD4+ T cells and absence of lymphocyte zones in spleens and mesenteric lymph nodes (83, 84). Polysaccharide A from *Bacteroides fragilis* was found to induce the Th1 response (83). Reduction of commensal microbiota in mice by using broad-spectrum antibiotics resulted in defective T and B cell responses against influenza infection (85). The findings that gut microbes can affect the immune functions, both locally and systematically suggest that bacterial species positively associated with PD-1 and PD-L1 blockade may enhance PD-1 and PD-L1 immunotherapy through regulation of the immune response. The previous study by Sivan et al. showed that *Bifidobacterium* species that inhibited tumor growth activated DCs, further supporting this view (70).

## The Possible Impact of Chronic Infections and Inflammation on PD-1 and PD-L1 Blockade Therapy

Several microbes cause chronic infections in humans, some of which are known to increase host PD-1 and PD-L1 expression (86–94). However, studies have not examined whether existing chronic infections in patients with cancer affect the efficacy of PD-1 and PD-L1 blockade therapy.

An example of a chronic infection is *Helicobacter pylori* infection. *H. pylori* are a Gram-negative bacterium that colonizes the stomach of more than 50% of the world population. While most of the individuals colonized with *H. pylori* are asymptomatic, some may develop chronic gastritis and peptic ulcers, and *H. pylori* colonization is also a risk factor for gastric cancer (95). Previous studies have shown that patients with *H. pylori* infection have a significantly higher level of pro-inflammatory cytokines, such as TNF-α (96–98). Das et al. showed that *H. pylori* increased the gastric epithelial expression of PD-L1 using a gastric epithelial cell line model (86). Furthermore, they showed that gastric epithelial cells exposed to *H. pylori* inhibited the proliferation of CD4+ T cells isolated from blood and the inhibitory effect can be blocked using antibodies PD-L1. Similarly, Wu et al. observed increased PD-L1 expression in gastric biopsies of individuals infected with *H. pylori*, and co-culture of *H. pylori* infected primary gastric epithelial cells with T cells isolated from blood induced T cell apoptosis (87). These results suggest that *H. pylori* infection may cause the non-specific inhibition of circulating T cells, including tumor-specific T cells. In addition to *H. pylori*, several viruses, such as the hepatitis B virus, hepatitis C virus, human papillomavirus, and Epstein–Barr virus are also able to establish chronic infections in humans and increase host PD-1 or PD-L1 expression (88–94). Future studies are needed to examine whether chronic infections or inflammation impact on the efficacy of PD-1 and PD-L1 blockade. A recent study by Kottke et al. using a mouse model showed that pro-inflammatory cytokine TNF-α promoted tumor recurrence, while TNF-α blockade prevented tumor recurrence (99–102). Some bacterial species that are known to reduce chronic inflammation after administration orally may be examined to see whether they can improve cancer treatment (103–108). If chronic infections or inflammation reduce the efficacy of PD-1 and PD-L1 blockade, it would be through mechanisms other than the induction of the PD-1 and PD-L1 expression in the tumor tissues, as previous studies observed better responses to PD-1 blockade in patients with higher expression of PD-L1 in tumor tissues (51).

## Future Directions

As discussed, despite the clear benefits of PD-1 and PD-L1 blockade in treating some cancer patients, the efficacy and the recurrence of tumor are issues that remain to be tackled. Emerging evidence suggests that modulation of the gut microbiota is a promising approach for improving PD-L1 and PD-1 blockade therapy. However, future studies are needed to further develop this research area.

The *Bifidobacterium* species, particularly *B. longum*, increased anti-PD-L1 efficacy in mice models and was positively associated with anti-PD-1 efficacy in metastatic melanoma patients. Future studies are needed to understand the molecular mechanisms of these *Bifidobacterium* species in enhancing PD-1 and PD-L1 blockade therapy. In addition to the *Bifidobacterium* species, various studies reported positive associations of gut microbes with PD-1 and PD-L1 blockade therapy at genus level. These microbes need to be identified at species and strain level and their potential anti-tumor mechanisms require further investigation.

Several bacterial and viral pathogens are known to cause chronic human infections and the pro-inflammatory cytokines are known to induce host PD-1 and PD-L1 expression. In addition, some of these pathogens are known to directly attach immune cells. Whether chronic infections caused by different pathogens impact on PD-1 and PD-L1 blockade therapy should be investigated, and appropriate strategies to enhance PD-1 and PD-L1 blockade therapy in these patients can then be developed accordingly. A suggested course of action is outlined in Table 3.

**Table 3:** Suggested future directions.

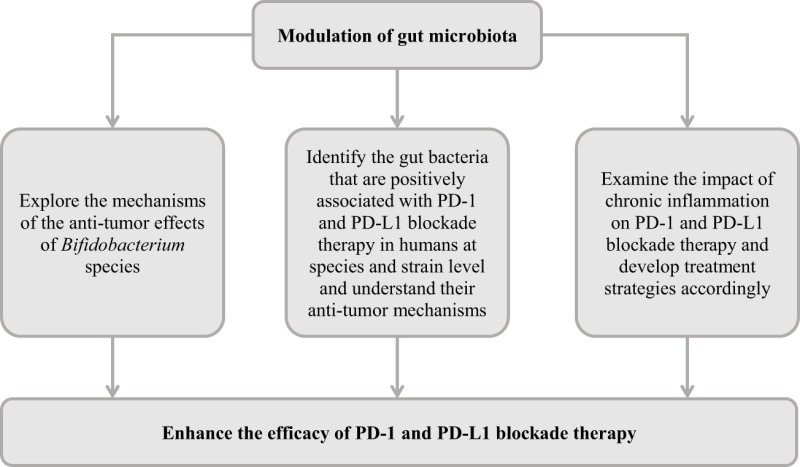

## Author Contributions

Wrote the paper: YW, LZ. Figures and tables: YW. Revised the paper: YW, LZ, RM, FL, SL. All authors have approved the final version of the manuscript.

## Conflict of Interest Statement

The authors declare that the research was conducted in the absence of any commercial or financial relationships that could be construed as a potential conflict of interest.
